# Synthesis of Resorcinol and Chlorophenol from Irradiation of 1,3-Dichlorobenzene in a Water Ice Environment by Low-Energy Electrons

**DOI:** 10.3390/ijms26020688

**Published:** 2025-01-15

**Authors:** Hassan Abdoul-Carime, Janina Kopyra

**Affiliations:** 1 Institut de Physique des 2 Infinis, Universite Claude Bernard Lyon 1, Universite de Lyon, CNRS/IN2P3, UMR5822, F-69622 Villeurbanne, France; 2 Faculty of Sciences, Siedlce University, 3 Maja 54, 08-110 Siedlce, Poland; janina.kopyra@uws.edu.pl

**Keywords:** synthesis of chlorophenol and resorcinol, 1,3-dichlorobenzene, water ice environment, low-energy (<12 eV) electrons, radical reactions

## Abstract

Dichlorobenzene is beneficial to industries, however, the release of this compound into the environment causes significant damage to ecosystems and human health, as it exhibits resistance to biodegradation. Here, we show that chlorophenol and resorcinol are synthesized from 1,3-dichlorobenzene in a water ice environment (1) directly on a poly-crystalline gold surface and (2) after low-energy (<12 eV) electron irradiation of admixture films. For the latter, at energies below 5.5 eV, the electrons solely decompose the chlorinated compound into radicals that further undergo reaction with surrounding water molecules. At higher energies (i.e., >5.5 eV) additional fragments, e.g., hydroxyl radicals, produced from the dissociation of water molecules, may also be involved in the chemistry. The present results may suggest strategies for potential eco-friendly, sustainable, and scalable processes for the mitigation of these halogenated compounds such as cold plasma and radiation, in which low-energy (<10 eV) electrons are predominantly produced.

## 1. Introduction

Halogenated organic compounds (HOCs) are widely used in industry as building blocks for the synthesis of insecticides [1], flame retardants, medicinal chemistry [2], or fabrication of polymers [3] since they are inexpensive to manufacture. Extensive use of these compounds led to the release of large quantities into the environment [4,5,6,7], and caused significant damage to the ecosystem/environment and human health. Thus, the elimination of the HOCs from the environment is becoming a challenge and several methodologies have been developed for this purpose (e.g., adsorption, microbiological, chemical, or photochemical).

Repurposing HOCs not only offers an alternative, desirable, and promising strategy for reducing environmental fingerprints [8,9], but also minimizes impacts by recycling these compounds. Reuse methods typically involve dehalogenation of the compound through activation of the halogen bond, i.e., the Cl-C bond, for the de-chlorination process. The removal of the chlorine atom from HOCs may be achieved by various different approaches, such as electrochemical reduction [10], chemical Williamson ester synthesis [11], nanoparticle catalysis [12], or by low-energy electrons. For the latter, these particles are abundantly produced with a distribution energy typically below 10 eV in eco-friendly synthesis technologies [13,14,15] such as radiation methods [16,17], low-energy electron beam [18], surface plasmon resonance [19], or cold plasma [20] processes. For instance, it has been already shown that the decomposition of chlorobenzene in a stream of air from a non-thermal (cold) plasma process leads to the synthesis of various byproducts containing a hydroxyphenyl group [21].

Dichlorobenzene, existing in three isomers (ortho-, meta-, and para-), is well known to have multiple industrial uses, such as manufacturing mothballs, lubricant, corrosion inhibitor, herbicides, deodorizers, or as air fresheners [7]. However, this compound is known to be an environmental pollutant, toxic, and persistent when released into the ecosystem, as the molecule appears to show resistance to biodegradation [22]. The present contribution studies the reactivity of 1,3-dichlorobenzene (1,3-DCB) or meta-DCB in a water ice environment under low-energy (<12 eV) electrons irradiation. Here we show the production of chlorophenol and resorcinol by reactions arising (1) already on the gold surface and (2) upon low-energy electron bombardment. These results can provide information on the degradation and, potentially, the reusability of dichlorobenzene through sustainable chemical synthesis techniques.

## 2. Results

Mass and temperature-programed desorption (TPD) spectra of vapor of the condensed 1,3-DCB, respectively, are shown in Figure S1. The gas phase analysis of 1,3-DCB molecules prior to deposition on cold substrate agrees with the ionisation mass spectrum from the National Institute of Standards and Technology (NIST) database [23], for which *m*/*z* 146 is the dominant species (100%, C_5_H_4_Cl_2_^+^), followed by *m*/*z* 111 (50%, C_6_H_4_Cl^+^). No species at *m*/*z* 110 and 128 are observed in the mass spectrum (Figure S1a). *M*/*z* 146 and 111 species observed from the TPD spectra of pure deposited 1,3-DCB on the poly-crystalline gold surface exhibit desorption peaks at 185 K accompanied with a small contribution at 250 K (Figure S1b). No other works on the desorption of 1,3-dichlorobenzene has been found in the literature for comparison. Nonetheless, it has been reported that the desorption peak temperature for various aromatic compounds from Au or Ag surfaces is in the range of 170–250 K (multilayer) and 230–310 K (monolayer) [24,25]; for instance, the desorption temperatures of chlorobenzene from Ag(111) are 170 K (multilayer) and 230 K (monolayer) [25].

**Figure 1:**
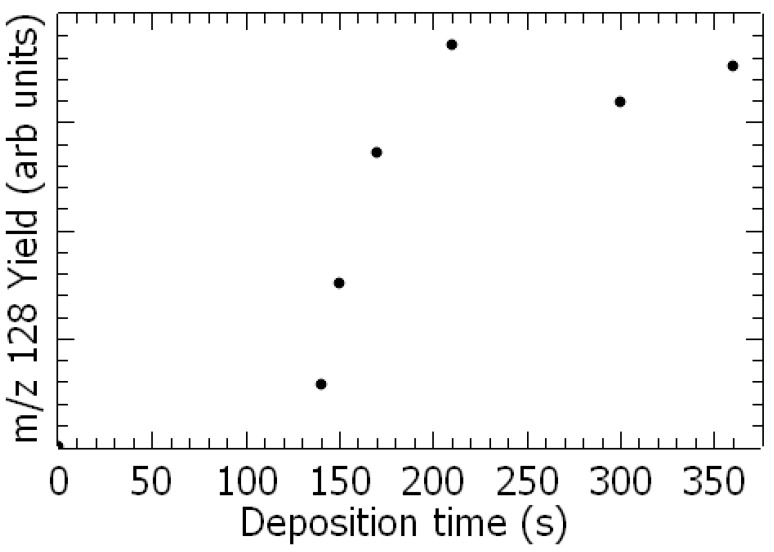
The *m*/*z* 128 yield as a function of the deposition time.

**Figure 2:**
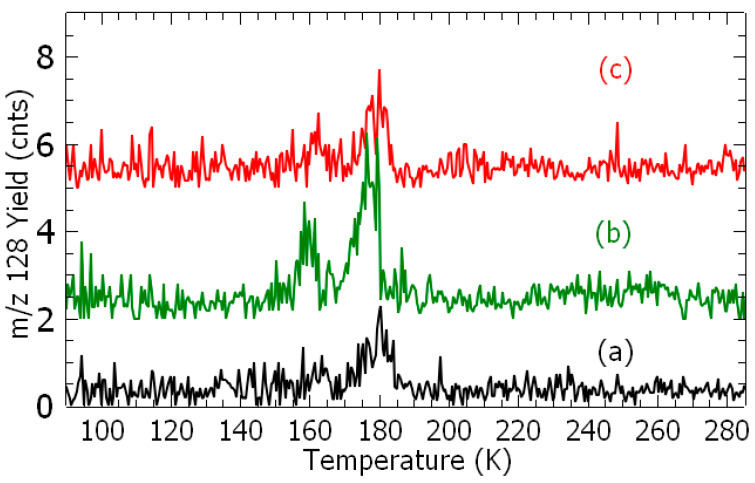

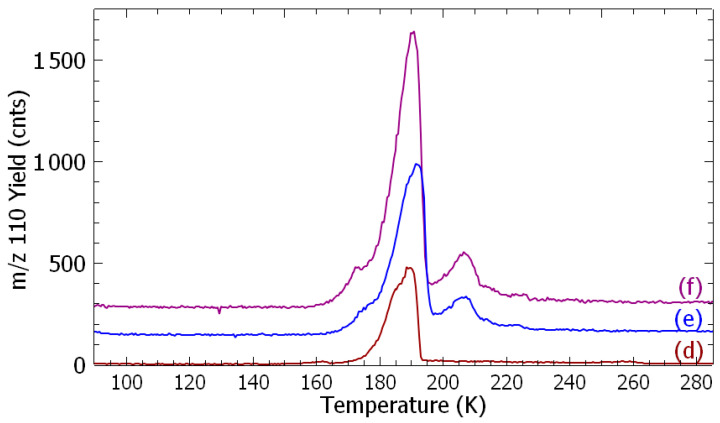
Temperature-programed desorption spectra: *m*/*z* 128 (chlorophenol) (a, black) no irradiation, (b, green) 1 eV at 2 nA, and (c, red) 7 eV at 29 nA; and *m*/*z* 110 (resorcinol) (d, brown) no irradiation, (e, blue) 4.0 eV at 0.73 nA, and (f, purple) 9 eV at 1.37 nA. Each sample was irradiated for 60 min.

After deposition of mixed water-1,3-DCB vapor onto the cold surface, TPD spectra already reveal the production of additional species at *m*/*z* 128 (Figure 1 and Figure 2 black) and 110, as shown in Figure 2 (brown), which can be assessed by their stoichiometry to chlorophenol (C_6_H_4_(OH)Cl) and resorcinol (C_6_H_4_(OH)_2_), respectively. The production of chlorophenol as a function of deposition time is exhibited in Figure 1. The yield increases linearly with deposition time until a plateau is reached. This result indicates that this species is produced at the metal surface, otherwise it would continuously increase with deposited time, i.e., thickness of the film.

Figure 2 presents TPD spectra of *m*/*z* 128 (a, b, and c) and 110 species (d, e, and f), respectively, recorded after the irradiation of fresh samples at different given electron energies for 60 min. For each spectrum, the integrated signal is subtracted from the contribution of species produced from the surface reaction (Figure 1) and normalized to the electron current (Figure S3).

**Figure 3:**
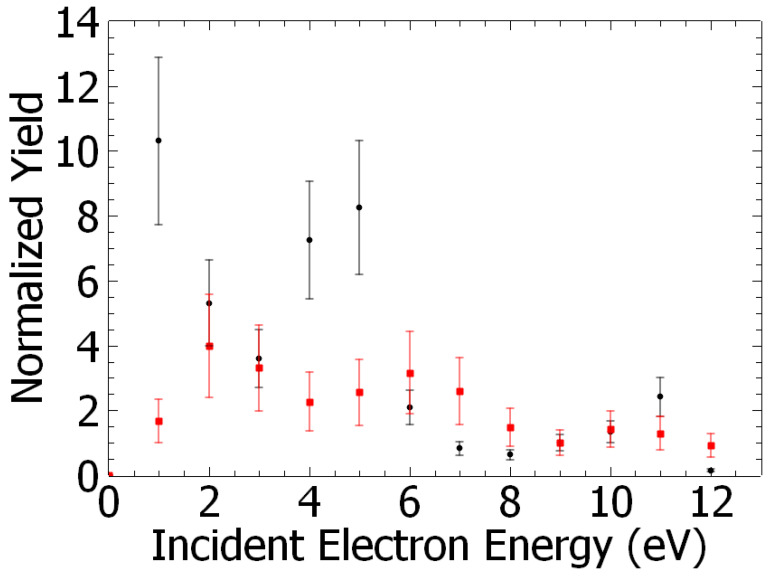
Chlorophenol (*m*/*z* 128, red squares) and resorcinol (*m*/*z* 110, black circles) yields as a function of the incident electron energy. Each point is an average of two to eight measurements, providing an accuracy of 25–40%. The yields are normalized to the yield value estimated at the electron energy of 9 eV. At this energy the measured yields are measured to be 5 cts/nA and 690 cnts/nA for chlorophenol and resorcinol, respectively. The 0 eV electron energy in this scale corresponds to the “no irradiation” yield taken as reference. The energy accuracy is about 0.5 eV (SI3).

The yields of *m*/*z* 128 and 110 species obtained by this normalization procedure are presented as a function of electron energy in Figure 3.

## 3. Discussion

As shown in Figure 1, chlorophenol can be produced from a reaction of the deposited admixture on a poly-crystalline gold substrate. Since H_2_O cannot be dissociated (i.e., via dehydrogenation) by the metal surface [26], the production of this new species must be triggered by the decomposition of adsorbed 1,3-DCB on the metal surface via the de-chlorination process. This has been reported for chlorobenzene on Pt(111) [27] or CCl4 on poly-crystalline gold [28] substrates. For dichlorobenzene, the available information concerns the 1,4-DCB isomer that is preferentially bound to the stepped Au(322) rather than the flat Au(111) surface, the Cl atoms being aligned with the step edge [29]. De-chlorination of dichlorobenzene (i.e., one or two chlorine atom removal) at the Si(100) [18] or Pd/Fe [30] surfaces producing chlorobenzene has been reported. The present observation suggests that de-chlorination of 1,3-DCB on the gold surface leads to a meta-chlorophenyl (or 3-C_6_H_4_Cl^⦁^) radical as an intermediate step. Indeed, no isomerization (e.g., transition of meta- to para-) is energetically accessible since the transition barrier between isomers is high (~2.8 eV) [31]. This produced meta-chlorophenyl radical can in fine undergo reaction with neighboring H_2_O molecules for the production of meta-chlorophenol, similarly to what has been reported for the reaction of the phenyl radical with water-yielding phenol [32]. The detected *m*/*z* 110 species (resorcinol) cannot be related to the cracking of 1,3-DCB in the mass spectrometer. Indeed, this measured *m*/*z* 110 yield would be about 5% of that of the *m*/*z* 111 species (SI2), representing a characteristic feature from the electron mass ionization spectrum of the precursor [23]. The TPD spectra in Figure S2 exhibit a weak feature at 154–157 K reminiscent of the desorption temperature of water from the poly-crystalline gold surface [33]. This observation suggests that the production of resorcinol arises from either (1) a sequential reaction 1,3-DCB ⟶ meta-chlorophenol, the latter undergoing a second de-chlorination on the gold surface followed by the reaction with water, or (2) a one-pot reaction consisting of a double de-chlorination of the precursor and reactions with water molecules. As the yield of meta-chlorophenol is lower than that of resorcinol by approximately 100 times, it suggests that process (1) is dominant. De-chlorination on a noble metal surface at a low temperature (<200 K) followed by complex surface chemistry has already been reported for Pt [34] or Au [26,28] surfaces.

After irradiating a freshly deposited admixture with electrons at given energies, TPD spectra exhibit an increase in both the meta-chlorophenol and resorcinol yields (Figure 2b,c,e,f). The desorption yield of meta-chlorophenol shows two peaks at T = 160 K and 180 K, and that of resorcinol three peaks at T = 173 K, 190 K, and 206 K. The 185 K and 190 K desorption temperatures may be associated to the expulsion of the synthesis products with 1,3-DCB from the film. The temperature of 173 K observed in the TPD spectrum of resorcinol coincides with that of the chlorobenzene ^27^, and the 206 K temperature is possibly related to a direct adsorption at the surface. Thus, the adsorbate becomes more strongly bound to the surface, leading to a “higher” desorption temperature.

The yields of meta-chlorophenol and resorcinol presented as a function of the incident electron energy are shown in Figure 3. They exhibit structures reminiscent of resonant processes. For instance, the yield function of meta-chlorophenol (Figure 3, red squares) shows peaks at about 2 eV, 6 eV, and 10 eV, and resorcinol (Figure 3, black circles) shows two distinguishable peaks at 1 eV and 5 eV. At these energies, dissociative electron attachment (DEA) effectively controls molecular fragmentation and the subsequent reaction [35]. Briefly, the colliding electron is trapped by the molecule to form a transitory negative ion, which further decays via dissociation forming an anion and at least one neutral species. For instance, it has been shown that the irradiation of water films with electrons at energies <10 eV electrons results in the syntheses of D_2_O_2_ [36] via a two-step process: (1) resonant dissociation of D_2_O into the D^−^ anion and the DO^⦁^ neutral radical [37], followed by an (2) OD + OD radical–radical reaction. In step (1), an impinging electron on the water film is captured by D_2_O to form a transitory anion (e.g., D_2_O^#−^) that decays into a negative fragment (D^−^) and its neutral OD^⦁^ radical counterpart. It is to be noted that DEA to D_2_O produces the anion at electron energies above ~5.5 eV with resonances observed at 7.2 eV, 9 eV, and 11 eV [38]. DEA to dichlorobenzene in different isomers has been investigated in the (0–2 eV) energy range [39], and the resonant production of the chlorine anion at 0.39 eV has been reported. A more recent DEA study, specifically of the 1,3-DCB isomer, extending the electron energy range to 10 eV, has shown that chlorine anion is resonantly produced, in addition to the low energy resonance, at “higher” energies (i.e., 2.3 eV, 3.65 eV, 5.69 eV, and 7.38 eV) [40]. Based on these data, we can suggest that at incident electron energies above 5 eV, the dissociation of both H_2_O and 1,3-DCB can produce the H^−^ and Cl^−^ anions and the respective associated neutral counterpart radicals, i.e., OH^⦁^ and C_6_H_4_Cl^⦁^. These radicals can then undergo reaction via, for instance, substitution of the Cl atom by the OH group [38] to form meta-chlorophenol. In the case of resorcinol, C_6_H_4_(OH)_2_, three possible scenarios can arise: (1) a two-electron dissociation process of 1,3-DCB to form a C_6_H_4_ bi-radical that further reacts with two OH^⦁^ radicals, and/or (2) DEA to meta-chlorophenol producing the C_6_H_4_(OH) radical with Cl^−^ anion, the former species reacting with a OH species, or (3) substitution of chlorine atoms by OH^⦁^ radicals on the benzene ring [29,41]. Thus, the yield function of the synthesized species (Figure 3) must be a convolution of those from the dissociation of H_2_O [36] and 1,3-DCB [37,39] induced by low-energy electrons. Electrons with energies below 5.5 eV cannot produce OH^⦁^ radicals by dissociation of H_2_O [36] as mentioned above. The production of meta-chlorophenol and resorcinol must then arise solely from the de-chlorination of 1,3-DCB by the electron (i.e., production of Cl^−^ anion) and further reactions of the formed radicals with water, similarly to those suggested above for the synthesis at the poly-crystalline gold surface, but within the irradiated film.

Analysis of the integrated energy yields of resorcinol vs. meta-chlorophenol indicates that the production of the former species by electrons is greater than that of the latter species by about two orders of magnitude (~140) (Figure 3). However, it was observed that the reaction at the electron energy below 5.5 eV (i.e., not implying dissociative electron attachment to H_2_O) is in general more favorable for the production of both C_6_H_4_(OH)_2_ and meta-C_6_H_4_(OH)Cl, however, by factors of 4 and 1.5, respectively, compared to the yields produced at electron energies above 5.5 eV. The yields of resorcinol and meta-chlorophenol can be compared for their production from surface reaction vs. after 60 min irradiation by a ~0.3 cm^2^ electron beam. Under the present experimental conditions, the irradiation of the films was found to generate approximately 6 and 4 times more C_6_H_4_(OH)_2_ and meta-C_6_H_4_(OH)Cl, respectively, than the surface reaction. We can also estimate the production of resorcinol vs. the deposited 1,3-DCB. The measured signal of the ‘ionized’ neutral species can be expressed as N^meas^ ~ N_mol_. σ, where N_mol_ represents the number of molecules entering the mass spectrometer and σ the 70 eV ionization cross section. Thus N_Res_/N_1,3DBC_ = (N_Res_/N_1,3DBC_)^meas^ (σ_Res_/σ_1,3DBC_). From (SI2), and by assuming σ_1,3-DCB_ and σ_Res_ to be similar to that of chlorobenzene and phenol [42], we found that at most, 20% of resorcinol can be synthesized from the surface reaction.

## 4. Materials and Methods

The experimental UHV (5 × 10^−10^ mbar) setup has been described previously [43]. The samples of dichlorobenzene and water (spectroscopic grade, SIGMA-Aldrich, St. Louis, MO, USA) were degassed by repeated freeze–pump–thaw cycles under vacuum before use. At room temperature, 1,3-DCB (~1.5 mbar) and H_2_O (23 mbar) vapors are introduced in this order, into a 27 mL mixing chamber prior to injection into the measurement chamber. The injected mixed (1.5:23) vapor is condensed onto an Au substrate, initially cryogenically cooled at 90 K by means of a closed-cycle He refrigerator. The substrate is resistively heated (>450 K) for cleaning prior to each molecule deposition step, and the temperature is measured using an E-type thermocouple (±2 K) fixed to the Au substrate. A volumetrically calibrated effusing gas quantity (MKS Baratron type 127) provides an estimate of the thickness of the film, which is estimated to be typically 5 monolayers, MLs, ± 40%. A pure water film of 1 ML would correspond to a thickness of 0.25 nm [41]. It is noteworthy that, in these experiments, the knowledge of the exact thickness of the film is not necessary, but the film must be sufficiently thick to avoid some possible effects of the metal substrate (i.e., quenching or enhancing process) and thin enough to minimize the multiple electron scattering (i.e., the mean free path of electrons in ice films is about 3 nm [44]) that would modify the energy of the reacting electrons. A monoenergetic electron beam (few tenths of nA, ~0.3 cm^2^), generated by the trochoidal electron monochromator that provides an energy selection of ΔE ~470 meV (Figure S3), impinges normally on the substrate for 60 min. The electron energy is calibrated by fast recording of the onset of the electron transmission curve [45]. This onset is rescaled every 30 s until there is no appreciable shift observed in the electron transmission curve since the charging of the film during the irradiation. Otherwise, the film would be irradiated at different electron energies, and thus the fragmentation process would consequently be modified, perhaps as well as the subsequent chemistry. After irradiation of the film, the substrate is heated at a rate of 12 K/min for thermal desorption. The desorbed neutral species are detected by recording the associated positive ions produced from 70 eV electron ionization of the molecules inside the quadrupole mass spectrometer (QMS, Balzer). The yield of “ionized” neutral species is recorded as a function of the substrate heating temperature by means of a temperature-programed desorption (TPD) spectrum. Each TPD spectrum is repeated 3 to 6 times, providing the accuracy of the measurements (c.a., ~40%).

## 5. Conclusions

Deposition of an admixture of 1,3-chlorobenzene-water on the poly-crystalline gold surface produces meta-chlorophenol and resorcinol from surface reactions. Irradiation of 1,3-dichlorobenzene in a water environment by low-energy (<12 eV) electrons also synthesizes these products, however, more abundantly resorcinol by about 30%. The synthesis operates via de-chlorination of the 1,3-chlorobenzene either on the gold surface or via dissociative electron attachment producing the Cl^−^ anion. The generated neutral radicals undergo further reaction with water molecules, or with OH radicals formed from the dissociation of H_2_O. However, the process implying dissociation of water molecules by electrons (i.e., at the energies above 5.5 eV) is not likely to be dominant.

The present study cannot be directly related to any yet scalable industrial processes since 1,3-DCB is in a water ice (90 K) environment. Nonetheless, the fundamental reactions for the production of chlorophenol and resorcinol are very likely to occur in a water-rich-environment. It is to be noted that the chlorophenol may be further deteriorated into phenol or resorcinol since the facile C-Cl bond dissociation is induced by low-energy electrons. This investigation may therefore open up perspectives for applications such as catalytic reaction with nanoparticles (Cu, Au, …) in water [12] (possibly with some side problem related to the regeneration of the catalyst metal atom) or cold plasma [21], for which dichlorobenzene may flow in a water-rich air stream for synthesis or at least the destruction of this contaminant.

## Data Availability

Data are contained within the article and Supplementary Materials.

## Supplementary Materials

The following supporting information can be downloaded at: https://www.mdpi.com/article/10.3390/ijms26020688/s1.
