# Supplementary material for: Synthesis of Resorcinol and Chlorophenol from Irradiation of 1,3-Dichlorobenzene in a Water Ice Environment by Low-Energy Electrons
Source: Int J Mol Sci. 2025 Jan 15;26(2):688. doi: 10.3390/ijms26020688 (PMC11766283; doi:10.3390/ijms26020688)

Supplementary Materials

Synthesis of Resorcinol and Chlorophenol from Irradiation of 1,3-Dichlorobenzene in a Water Ice Environment by Low-Energy Electrons

Hassan Abdoul-Carime [1,*] and Janina Kopyra [2]

[1] Institut de Physique des 2 Infinis, Universite Claude Bernard Lyon 1, Universite de Lyon, CNRS/IN2P3, UMR5822, F-69622 Villeurbanne, France
[2] Faculty of Sciences, Siedlce University, 3 Maja 54, 08-110 Siedlce, Poland; janina.kopyra@uws.edu.pl
* Correspondence: hcarime@ipnl.in2p3.fr

Figure S1 : Mass and temperature desorption spectra of pure 1,3-dichlorobenzene.

(a) Mass spectrum of 1,3-dichlorobenzene in the [70-150] amu range. The peaks observed at m/z of [73-76], [84-85], 111 and 146 (with the associated ^{37}Cl) agree with the NIST mass spectrum [ref.]. The dashed blue lines indicate the m/z position of resorcinol (m/z 110) and chlorophenol (m/z 128).

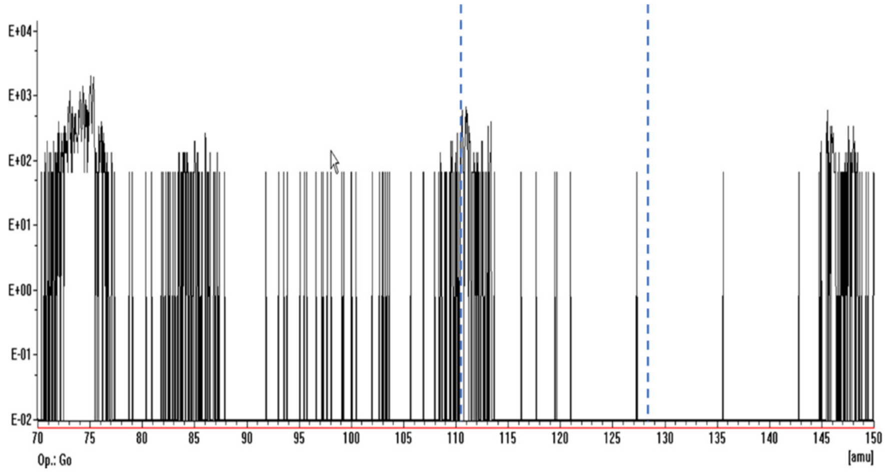

(b) TPD spectrum recorded at m/z 146 (black) and 111 (orange). The species are attributed to the ionized 1,3-dichlorobenzene parent, and the fragment ion produced inside the quadrupole mass spectrometer.

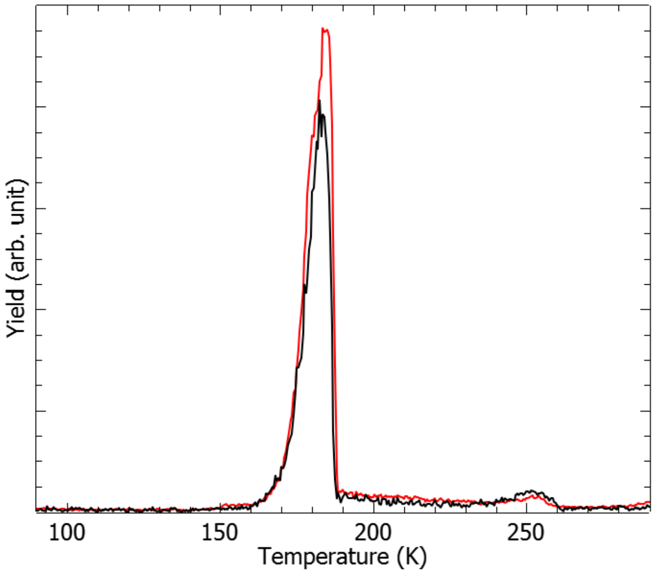

Figure S2 : Temperature Programmed Desorption spectrum of (a) m/z 111 (orange) and (b) m/z 110 (black) species. From the NIST database, the ionization of 1,3-dichlorobenzene leads to production of m/z 111 ($C_6H_4Cl^+$) as the cracking of the precursor, but also less than 5% of m/z 110. Here, the comparative yield of m/z 111 and 110 species clearly indicate that the latter species does not arise from the precursor fragmentation in the ionization mass spectrometer.

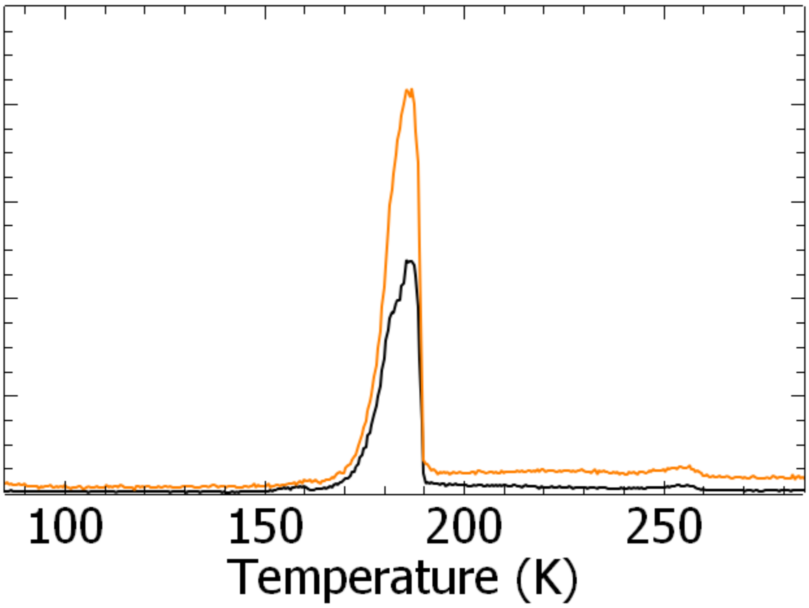

Figure S3 : Transmitted current (solid black) and dI/dE (dashed red). From the derivative as the function of the Electron Accelerating Voltage (V). The onset in the transmitted current curve, E_0, provides the « 0 eV » electron energy and, the full width half max of the dI/dE curve, the energy resolution. Note that the gain in the energy resolution comes at the expense of the electron current.

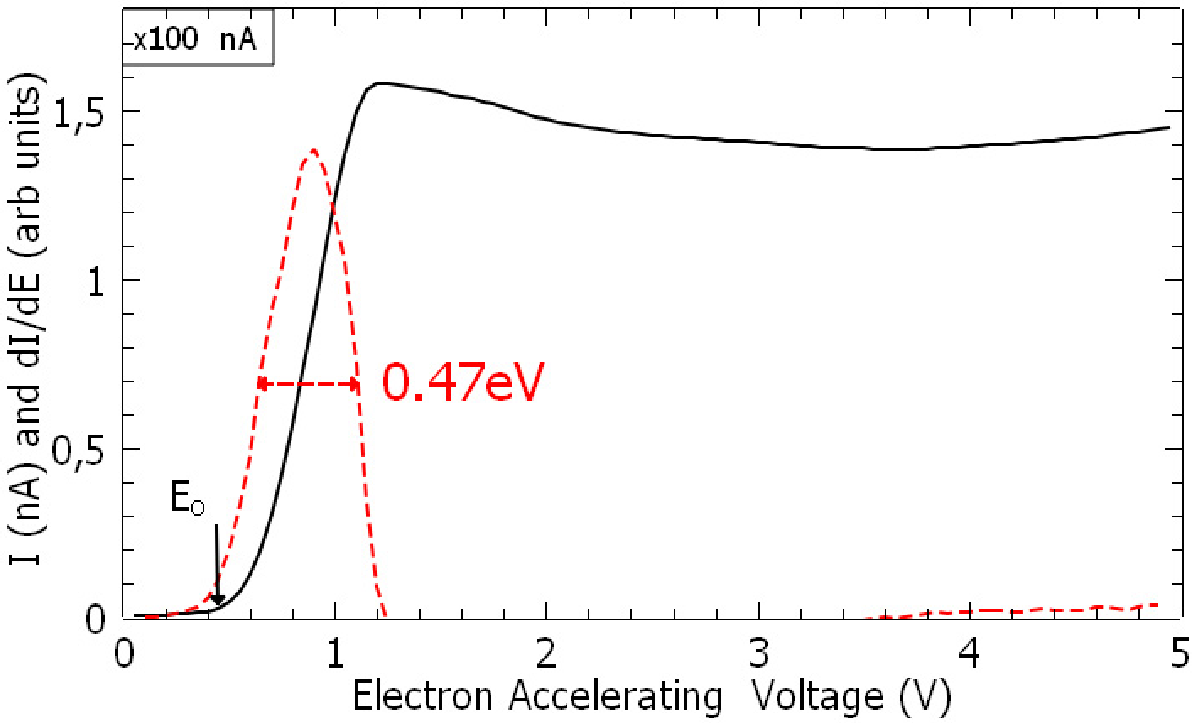

Supplement: Supplementary file 1 [file ijms-26-00688-s001.zip › ijms-3397599-supplementary.pdf]
